# Simulating the behavior of patients who leave a public hospital emergency department without being seen by a physician: a cellular automaton and agent-based framework

**DOI:** 10.1590/1414-431X20176961

**Published:** 2018-01-11

**Authors:** Milad Yousefi, Moslem Yousefi, F.S. Fogliatto, R.P.M. Ferreira, J.H. Kim

**Affiliations:** 1 Departamento de Engenharia de Produção e Transportes, Universidade Federal do Rio Grande do Sul, Porto Alegre, RS, Brasil; 2 Department of Mechanical Engineering, Roudehen Branch, Islamic Azad University, Roudehen, Iran; 3 Departamento de Engenharia Mecânica, Universidade Federal de Minas Gerais, Belo Horizonte, MG, Brasil; 4 School of Civil, Environmental and Architectural Engineering, Korea University, Seoul, Republic of Korea

**Keywords:** LWBS, Emergency department, Agent based simulation, Cellular automata, Healthcare industry

## Abstract

The objective of this study was to develop an agent based modeling (ABM) framework to simulate the behavior of patients who leave a public hospital emergency department (ED) without being seen (LWBS). In doing so, the study complements computer modeling and cellular automata (CA) techniques to simulate the behavior of patients in an ED. After verifying and validating the model by comparing it with data from a real case study, the significance of four preventive policies including increasing number of triage nurses, fast-track treatment, increasing the waiting room capacity and reducing treatment time were investigated by utilizing ordinary least squares regression. After applying the preventing policies in ED, an average of 42.14% reduction in the number of patients who leave without being seen and 6.05% reduction in the average length of stay (LOS) of patients was reported. This study is the first to apply CA in an ED simulation. Comparing the average LOS before and after applying CA with actual times from emergency department information system showed an 11% improvement. The simulation results indicated that the most effective approach to reduce the rate of LWBS is applying fast-track treatment. The ABM approach represents a flexible tool that can be constructed to reflect any given environment. It is also a support system for decision-makers to assess the relative impact of control strategies.

## Introduction

An important key performance indicator in emergency departments (EDs) is the number of patients who leave the ED without receiving treatment. These patients, though partially using the resources of ED and making the department crowded, might decide to leave before being seen (LWBS) because of long waiting time or queues. A study by Fry et al. (1) reported that 70% of LWBS patients return to EDs in the following 24 h while 11% of them require hospitalization within seven days of their initial visit (2).

Several studies had focused on the impact of different factors on LWBS and the time that patients wait before they leave. Weis et al. (3) found a correlation of 0.665 between the number of LWBS patients and National Emergency Department Overcrowding Scale (NEDOCS). To do so, they studied 214 2-h periods. NEDOCS categorizes EDs in 6 different groups ranging from "not busy" to "dangerously overcrowded" based on different items. More details on NEDOCS can be seen in (4).

Computer based simulation tools are well-known models to study the behavior of hospitals in general and EDs and their sub-systems and processes in particular. In order to do these analyzes, the results of several so-called "what-if" scenarios are compared. "What if" scenarios enable the decision-makers to analyze the response of a system to potential changes.

The behavior of impatient costumers has been the topic of interest in many researches. Boots and Tijm (5) proposed a model in a call center to study the behavior of callers who give up after waiting for more than 20 s. Whitt (6) provides an approximating algorithm to measure the performance of the basic call center queuing model (M/GI/s/r + GI) with a Poisson arrival process to predict abandonment in call center customers.

Wiler et al. (7) modified the same model and queuing theory to apply it in an ED, using a Weibull distribution to estimate the tolerance time of patients in ED. Although these studies considered some variables including capacity, number of arrivals and service time, the interactions among customers/patients and their effect on the systems were neglected. Computer simulations can be categorized according to different criteria. In one category, the simulation studies are divided into discrete event simulation (DES) and agent based simulation (ABS) groups. Each group has its own advantages and disadvantages and both are used in healthcare industry. For instance, a study of Duguay and Chetouane (8) models an ED using DES and their results show that the DES tool can effectively simulate the complexity of healthcare industry in an ED in Canada. They also recommend that a combination of total quality management and continuous improvement techniques be used in collaboration with DES. To improve the performance of their case study, first they evaluated the existing condition of the ED through data collection and by studying different variables including number of nurses, number of doctors and number of beds. Afterwards, by analyzing the waiting times and different scenarios in terms of number of staff in each section and also varying number of examination rooms in the predefined budget limits, they were able to improve the performance of their ED case study. A relatively recent literature review by Gul and Guneri (9) investigated both DES and ABS studies conducted in healthcare industry. They showed that although DES are the most commonly used tools in healthcare simulation, a recent increase in using ABS as a tool to simulate EDs has been observed.

In recent years, attention to machine learning techniques and computer simulations in healthcare industry have increased (10,11). Pan et al. (12) provided an agent based framework for simulating social and human behaviors during emergency evacuations. Yousefi et al. (13) developed a generalized ABM to simulate EDs. Their model was implemented using NetLogo platform and they claim that the proposed approach can be applied to different EDs with some minor modifications. Yousefi and Ferreira (14) introduced an ABM combined with group decision-making technique to improve the performance of an ED considering various key performance indicator including LWBS, LOS, waiting time, number of deaths, number of wrongly discharged patients and total number of discharged patients. In their study, patients decide to continue the treatment or LWBS based on only one factor, which is length of waiting time.

The main objective of this study was to simulate the behavior of patients who LWBS considering their own experiences as well as their interactions with other patients in the ED. To incorporate the complex dynamic behavior of patients into the simulation, a framework based on ABS and cellular automata (CA) technique is introduced and validated using data available from an ED in a public hospital in Belo Horizonte, capital of the Brazilian state of Minas Gerais.

**Figure 1.**
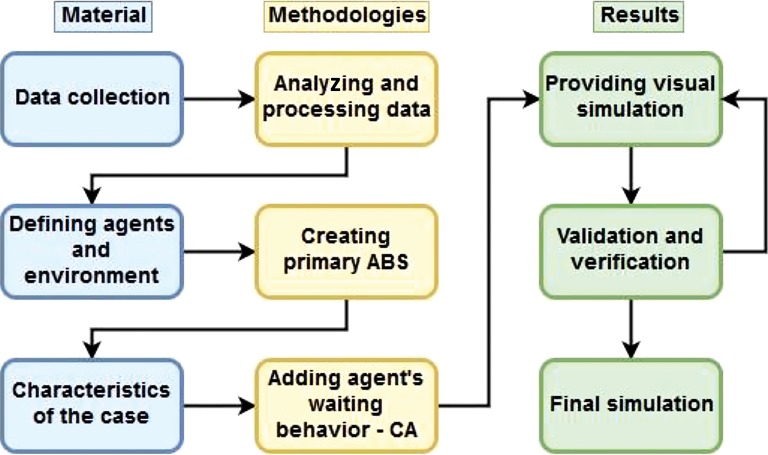
Simulation framework. ABS: agent based simulation; CA: cellular automata.

The introduced framework, while mainly focusing on the behavior of the LWBS patients, could be generally employed in any other healthcare simulation with minor modifications. Figure 1 demonstrates the framework of this study including the material, applied methodologies and results.

## Material and Methods

### Agent based simulation

Although DES is mostly used to simulate the flow of patients in EDs and for finding bottlenecks in a short time, this approach has its limitations when it comes to human behavior and interactions among different components of the simulation. In an ED, most of the principal agents are human; therefore, they interact with each other and learn not only from their own experiences but also from the experiences of people around them. The treatment process of a patient in an ED is nothing but the result of interactions between an agent (patient) with other agents (e.g., receptionist, doctor and nurse). In a DES, the possible path of an entity (components of a DES system) is pre-determined, which makes this approach simple and hence, limited in decision-making (15). Therefore, in this study, an ABS is utilized to simulate human behavior and interactions between people and the environment.

**Figure 2.**
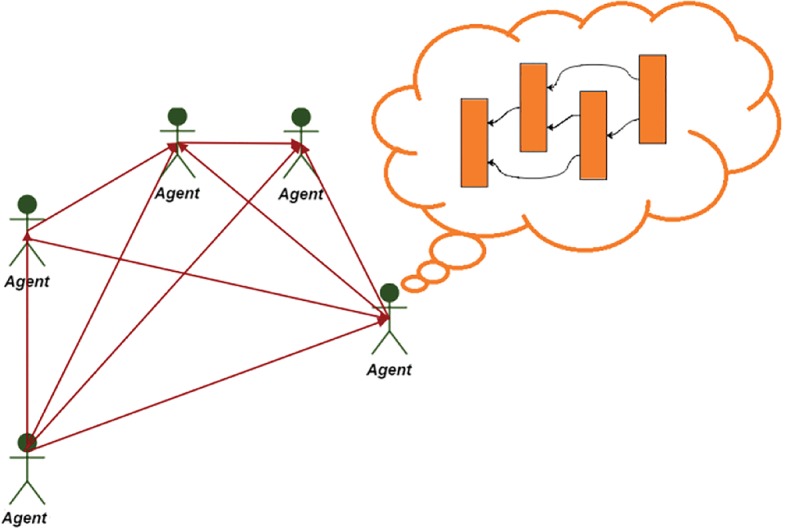
Agent based simulation system. Lines represent the interactions between agents and orange rectangles represent the decision-making process of each agent.

An ABS system contains a group of autonomous individuals that interact with each other yet make their decisions independently. These individuals are known as agents. A typical ABS consists of three main components: 1) agents and their characteristics and behavior; 2) relationships between agents and their interactions; and 3) environment (Figure 2). In this figure, each line represents the interactions between agents and orange rectangles represent the decision-making process of each agent.

Each simulation could potentially have various types of agents and each type have different number of agents. Although the focus of this study is on patients, there are other agents in the simulation including doctors, nurses and receptionists. Each of these agents, while having their own distinct behaviors, continuously interact with other agents. In this study, NetLogo 6.0.1 (16), an open source ABS platform, was used to simulate agents and their behaviors in an ED. NetLogo has been successfully employed in simulating complex systems such as for modeling the immune system (17), a distributed intelligent traffic system (18) and soil organic matter (19).

**Figure 3.**
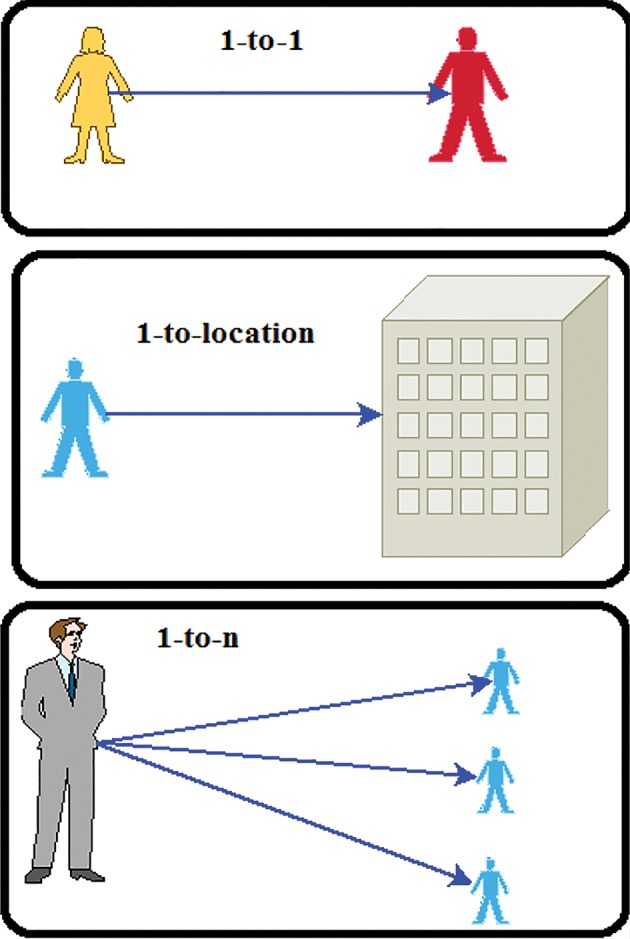
Different types of agents' communications.

Communication is defined as an output of any agent, which could be received by other agents as well as locations. Based on these communications, an agent might change its state or as well continue with the same state. Figure 3 demonstrates the three types of communications used in this study, which can be described as follows.

#### One-to-one communication

This type of communication happens between two individual agents when a message is delivered exclusively from one to another. While this message could potentially change the state of the receiver, it might as well go without an immediate effect. For instance, when a patient goes to a triage room a message goes to a triage nurse and changes its state from "waiting for a patient" to "giving service".

#### One-to-n communication

In this type of communication, one sender sends a similar message to a group of agents. When a nurse informs a group of patients to go to another section, the type of communication is one-to-n.

#### One-to-location communication

This type of communication happens when an agent sends a message to all agents in a specific location. For instance, the communication between a nurse and all patients in a waiting room informing them of unavailability of beds is categorized as a one-to-location type. This message is sent by a single sender and will be received by a group of agents who are in that waiting room (20).

Another type of communication that is classified under "one-to-location communication" is when a message is transferred to a place during a period of time and hence will be received by any agent that happens to go to that location during this specific time period.

**Figure 4.**
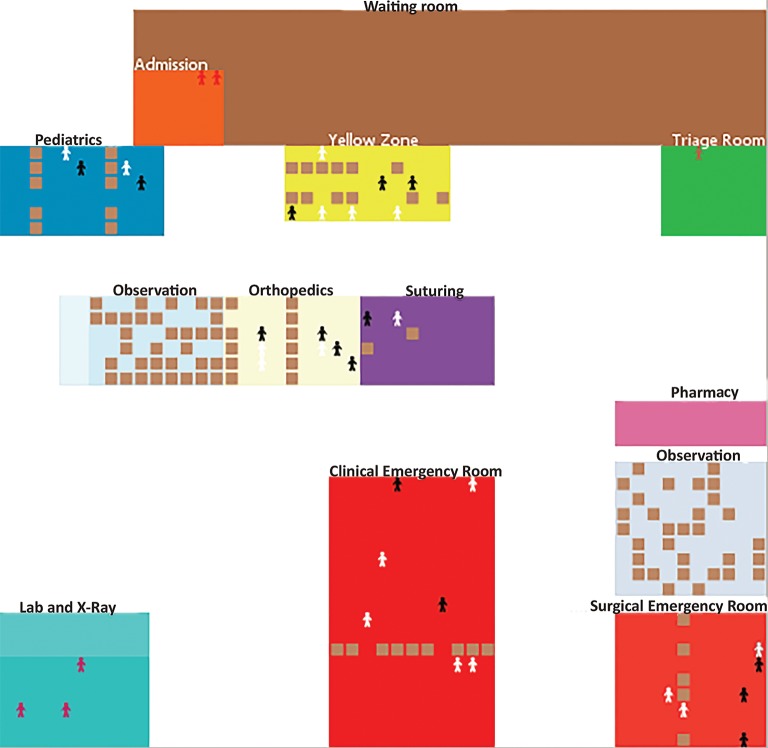
Environment of an agent-based simulation system of an emergency room produced by NetLogo. Each section of an emergency department is represented by a distinct color and each small square represents a bed. Doctors and nurse technicians are shown in black and white, respectively.

In ABS, both on micro and macro levels, agents are interacting in a shared environment that increases their behavior's diversity. Agents display unique behaviors because of their different interactions with this environment. Figure 4 shows the environment in this ABS where each section of an ED is represented by a distinct color and each small square represents a bed. Doctors and nurse technicians are shown in black and white, respectively.

**Figure 5.**
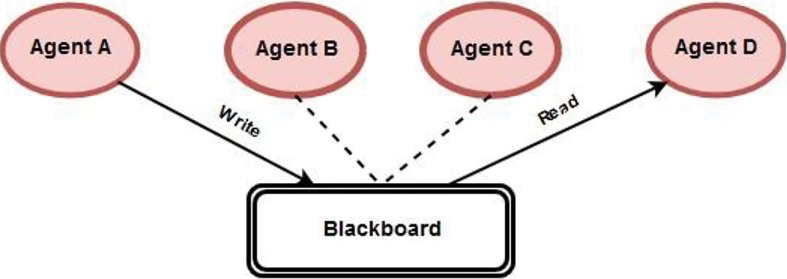
Schematic diagram of a simple blackboard system in agent based simulation.

In this study, a simple blackboard system is implemented for interactions between agents in general and decision making specifically. A blackboard system contains a shared database area for the whole ABS that is surrounded by the information from agents (Figure 5). All agents are able to read from and write on this blackboard. Consequently, at each step of the simulation all agents will be aware of the changes and make their decisions based on this shared information. The blackboard system works as a black box for the simulation platform (21). The blackboard system helps the agents to be updated about the number of patients who are waiting in the waiting room as well as the history of patients who have left the ED.

### Cellular automata (CA)

In this study, different tools were used to simulate the human behavior. One of the most useful approaches in simulating queues is CA. Although the initial applications of CA in the early 1950s were in the field of biological systems, the expansions of CA were introduced in "A New Kind of Science" by Wolfarm later in the early 1980's (22). CA are mathematical methods for representing complex models when the interactions between agents are based on local rules. Wolfarm (22) used a simple one-dimensional CA where each cell has one of the options "ON" or "OFF" with 4 neighbors, 1 at right, 1 at left, 1 in front and 1 at the back side. Although various applications of CA can be found in queuing problems (23,24), to the best of our knowledge, this is the first application of CA in an ED simulation.

In this study, a one-dimensional CA with 2 behavioral parameters (a, b) was employed. Generally, a ring structure is used in queuing problems where it is considered that each agent has one agent in front and one at the back. Unlike usual queues, in our study the agents did not line up to wait for their service. Instead, they sit in a waiting room to be called by a receptionist or a nurse. Therefore, to better simulate the behavior of patients in the real world and make the calculations simple, we assume that at any given time each agent can have up to four agents around it.

**Figure 6.**
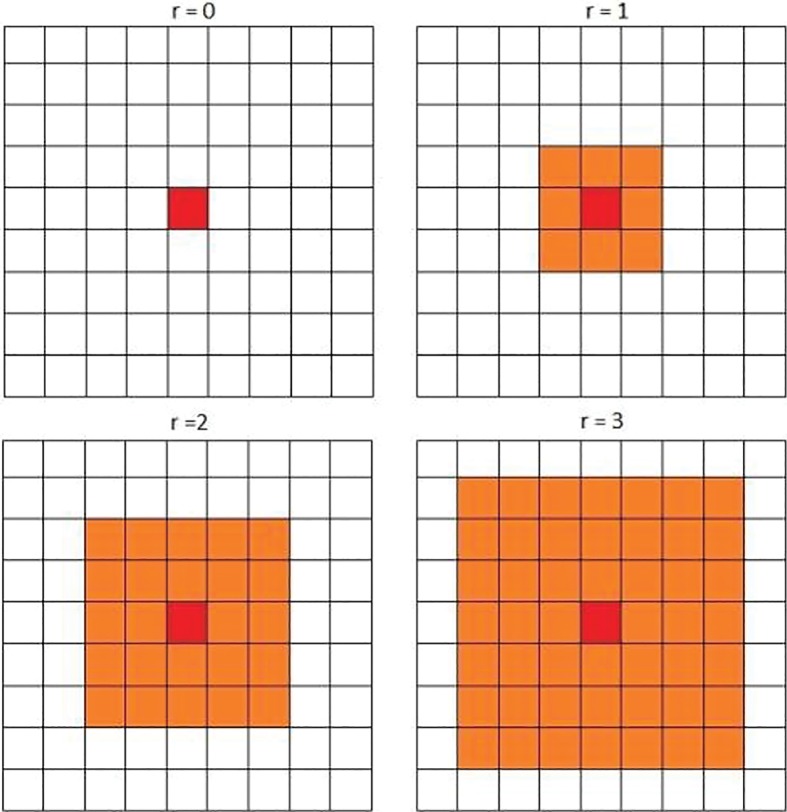
Moore neighborhood for r=0, r =1, r=2, and r=3. r: range.

**Figure 7.**
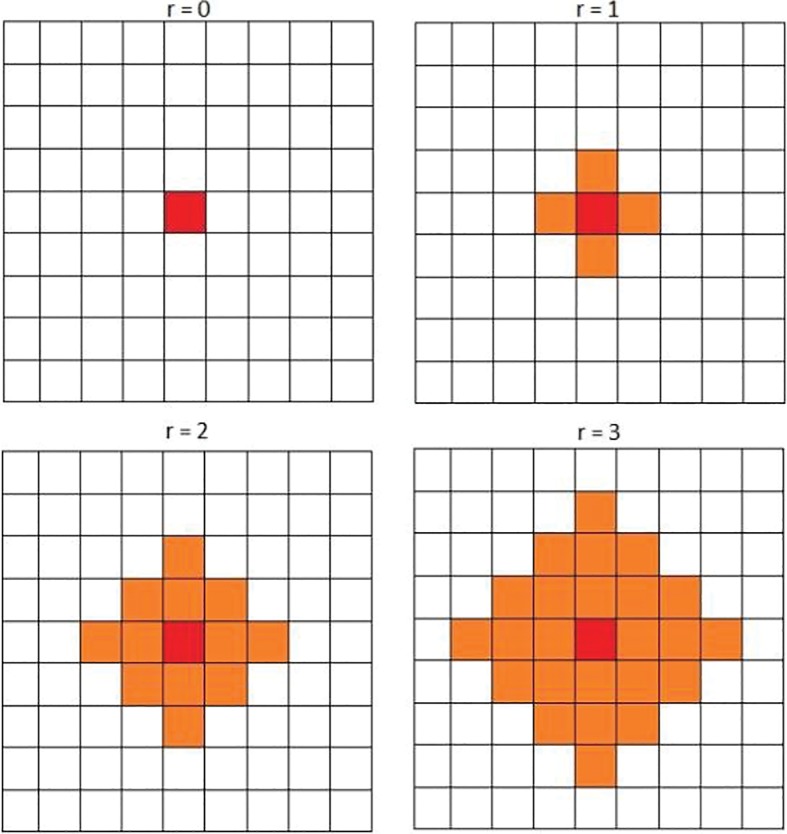
Neumann neighborhood for r =0, r=1, r=2, and r=3. r: range.

In general, there are 2 well-known methods for defining the neighborhood of an agent in CA, the Moore and Neumann neighborhoods. As can be seen in Figure 6, the Moore method has a squared shape, while the Neumann, for neighborhood ranges of 0, 1, 2, and 3, is diamond shaped (Figure 7). CA divides the environment into cells where the neighborhood of each cell at (x_0_, y_0_), for the neighborhood range of r, is defined as follow:

(Eq. 1) $$N_{{\text{x}}_{0}}^{\text{M}}{}_{y_{0}}=\left\{\left(\text{x, y}\right):\;|\text{x}-{\text{x}}_{0}|\leq \text{r}\right\}$$

(Eq. 2) $$N_{{\text{x}}_{0}}^{\text{M}}{}_{y_{0}}=\left\{\left(\text{x, y}\right):\;|\text{y}-{\text{y}}_{0}|\leq \text{r}\right\}$$

As mentioned earlier, the adopted CA in this study have two behavioral parameters (a, b) that make the agents decide whether or not to keep waiting in the ED. These parameters are as follows: a) agent's memory, that is the time that the agent waits in ED, and b) Neighbors' experience.

In this study, first we extracted the estimated tolerance time from the literature. Shaikh et al. (25) conducted their study from March to July 2010 and studied the behavior of 375 participants considering their age, gender, acuity level, race and insurance status. Data analyses showed that 51% of patients wait up to 120 min before leaving the ED, 17% wait 120 to 480 min, and the remaining 32% wait indefinitely to receive a treatment. In our simulation, patients do not leave the ED only based on their own memory; they also have a look at their neighbors. Therefore, we define "a" as tolerance time. When an agent reaches its tolerance time, it starts communicating with its neighbors (if any) and memorizes the whole process. When a patient reaches his tolerance time and did not have neighbors since his arrival, which means N=0 at all times, he leaves the ED at their tolerance time.

Patients with N≠0 neighbors will check their memory and leave the ED should N_delayed_≥N_normal_. In the case of N_delayed_<N_normal_, the patient will wait for an additional T minutes to receive the treatment.

(Eq. 3) T=0.1*ToleranceTime

If a patient does not receive treatment after the additional time, they will leave the ED.

In this study, the level of ED overcrowding is calculated similar to NEDOCS (4) by using an online platform. NEDOCS index varies from 0 (not busy) to 200 (dangerously overcrowded). In this framework, by increasing the NEDOCS, the tolerance time of patients decreases while it could increase by 30% of the normal tolerance time when the ED is not dangerously overcrowded.

### Case study

**Figure 8.**
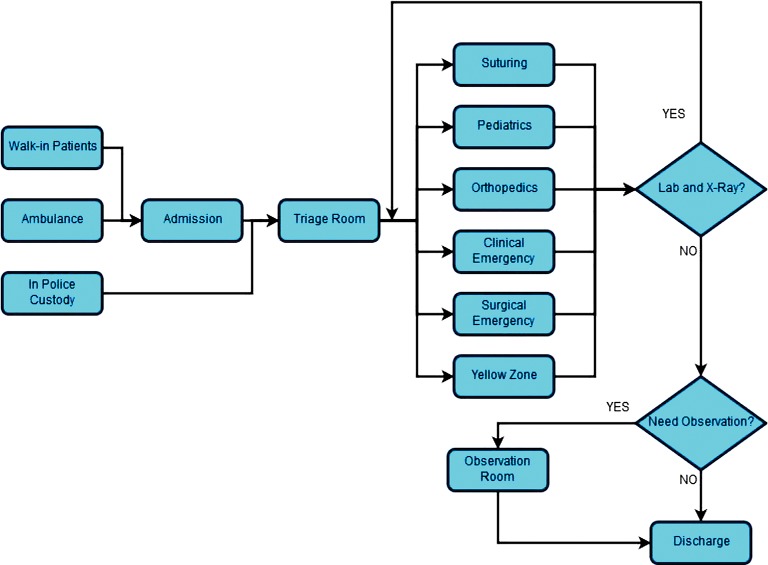
Flow of patients at an emergency department.

In this study, an ED in Hospital Risoleta Tolentino Neves (HRTN), a tertiary hospital located in the capital of Brazilian State of Minas Gerais, Belo Horizonte, was studied for the evaluation of the proposed method. This ED, which operates 24/7 and receives 162 patients in a day on average, contains various sections including suturing, orthopedics, pediatrics, clinical emergency room, surgical emergency room and yellow zone. Figure 8 demonstrates the flow of patients in HRTN. The patient arrival to the ED is a non-homogenous Poisson process with a rate λ(t) and 24 intervals. Patients may arrive to the ED by themselves, in custody of police or by ambulance. Patients start the procedure by going to the admission to provide their information to a receptionist. Patients in police custody skip the admission and got directly to the triage room. In the triage room, a triage nurse checks the acuity of patients based on Manchester triage system (MTS) to categorize them in one of the five MTS categories ranging from the most urgent (category one) to the least urgent (category five).

Following triage, patients wait in a waiting room to be called based on their categories and availability of the section required. If a patient needs diagnostic services such as laboratory or X-ray exams, they will proceed to the respective section. The patient will return to their initially assigned section and resume their treatment afterwards. Generally, the treatment will result in either the patient's discharge or hospitalization.

As mentioned before, the focus of this study is primarily the patients who LWBS, a factor that has been neglected in most of the previous ED simulation studies (14). In this model patients may decide to leave the hospital at any stage of their treatment.

## Results

At the beginning of the simulation, no patient is in the ED, therefore, the obtained results from this period might not represent the real condition. While the simulation is run for 3 consecutive days (4320 min), to avoid any bias at the beginning of the simulation, the data collection starts after 2 days (2880 minutes) which means that the first 2 days were set as the warm-up period.

Initially, the simulation model needs to be verified and validated to make sure that not only it correctly represents the actual patient flow in the hospital but also it produces reliable results. Firstly, the verification was qualitatively performed by comparing the created animation with the ED routine. The hospital manager and district coordinator of the ED also verified the simulation model. Secondly, the simulation model was quantitatively validated for the total time that patients spent in the hospital and the weekly number of discharged patients. This data was extracted from the emergency department information system (EDIS) in the first half of 2016.

The simulation results were compared with this extracted data. As can be seen in Table 1 with confidence level of 95% (a=0.05), there was no significant difference between the results obtained from the simulation model and the real data from EDIS. Moreover, the number of discharged patients in a week from the simulation and the real case was compared. The throughput for the real system was 1120 while the throughput of the simulation was 1108.

**Table 1.** Comparison of length of stay from the simulation model and the emergency department information system.

Section	Actual time (min)	Simulation time (min)	Confidence interval (95%)
Pediatrics	310.72	280.25	(286.65–342.54)
Yellow zone	412.46	389.45	(361.10–429.92)
Orthopedics	180.45	189.35	(177.32–199.47)
Surgical emergency	438.76	412.16	(401.21–471.52)
Clinical emergency	487.41	472.01	(462.12–513.68)
Suturing	196.45	189.42	(180.35–212.25)

In order to see the effect of CA approach in simulating behavior of those who LWBS, the simulation model is executed 30 times with LWBS variable and another 30 times without LWBS. The number 30 was selected based on a *t*-test that proved that there was no significant difference in the averages of two data sets when each model is executed 30 times.

Comparing the average LOS of each data set with actual times from EDIS shows that applying the LWBS approach in the simulation results in 11% improvement. On the other hand, our simulation results indicate that the rate of LWBS varies between 8.64 to 10.12%, which is close to the data from our case study.

Having been verified and validated, the simulation was then employed to evaluate the effect of different preventive policies on the LWBS rate. Four policies were extracted from reported case studies, focused on curbing rising LWBS rates, and by interviewing experts. To study the impact of each policy and analyze the data set, a simple ordinary least squares (OLS) regression was applied where rate of patients who LWBS in a day was considered the dependent variable. The obtained results from OLS show that all policies are significant at the level of 5%. A brief explanation of these policies is as follows.

### Policy 1: Increasing the number of triage nurses

In the current resource planning of the ED only one triage nurse is in charge of triaging. In this policy, another triage nurse would be added to the triage room.

### Policy 2: Fast-track treatment

In this approach, a doctor will treat patients with least urgent conditions (MTS categories four and five) right after triage room. In the current situation, these patients have to wait longer than other patients to be attended.

### Policy 3: Increasing the waiting room capacity

In this case, the size of the waiting room is assumed to increase from 30 to 40.

### Policy 4: Reducing treatment time

In this policy, it is assumed that the hospital management would train their doctors and nurses in a way that and they could perform their assigned tasks 10% faster than before. It should be noted that the decrease in the total LOS will be less than 10%.

Table 2 provides the LOS and rate of LWBS for baseline and when each policy is applied to the simulation. The improvement in LOS and LWBS is also shown for each policy. Although all policies have shown improvement in LWBS, the fast-track treatment outperforms other policies in terms of decreasing LWBS by 60.87% and improving the average LOS by 11.92%. Increasing the waiting room capacity did not improve the LOS, as expected, yet an improvement of 13.58% in the number of patients who LWBS is noticed due to decreasing the overcrowding score of the ED.

**Table 2.** Comparing the impact of preventive policies on LOS and LWBS.

Scenarios	LOS (min)	LWBS (%)	Improvement in LOS (%)	Improvement in LWBS (%)
Baseline	285	10.53	–	–
Policy 1	270	5.01	5.26	52.42
Policy 2	251	4,12	11.92	60.87
Policy 3	289	9.10	-1.40	13.58
Policy 4	261	6.14	8.42	41.69

LOS: length of stay; LWBS: leave without being seen.

## Discussion

To the best of our knowledge, this study provides the most comprehensive simulation model of LWBS patients by a combination of ABS and CA. In the proposed simulation model, not only the patients' own memory in ED is considered but also, by incorporating CA, the patients are able to interact with other patients around them in order to make a decision on whether to wait longer or LWBS.

Furthermore, NEDOCS index was used to calculate the level of ED overcrowding, which is another factor that affects patients' decisions. To validate the simulation model, the results from the simulation was compared to the available data from an ED. After validation and verification of the simulation model, four preventing policies were applied to the model to investigate their impact on LWBS.

Although an OLS regression showed that all policies significantly improved the patient flow in terms of LOS and LWBS rate, the fast-track treatment policy was better than the others. In this policy, a doctor is assigned to treat patients categorized as four and five based on the MTS, and unlike the normal procedure, patients with least severe problems do not face long waiting times to be attended.

This study emphasizes interactions between patients in the ED to study the behavior of those who leave the hospital unattended. The proposed model provides a tool to study the impact of changes in ED on LWBS without interrupting the routine of the hospital. In this paper three factors of personal memory, neighbors' experiences and overcrowding scale were considered. As limitations, only one public ED in Brazil was included and the data collection on tolerance time of different types of patients were not performed.

New decision-making factors can be added to the model in future studies. More importantly, the same model, can be effectively applied in other EDs. The proposed CA approach can be applied to different problems in hospitals including investigating the spread of viruses, transmissible diseases and infections. Moreover, the CA application can be a useful tool to find better layouts in EDs based on different criteria.
